# Therapy-induced cardiotoxicity in drug-resistant tuberculosis patients: A systematic review and meta-analysis

**DOI:** 10.21542/gcsp.2025.53

**Published:** 2025-10-31

**Authors:** Lies Dina Liastuti, Kelvin Kohar, Haifa Mayang Lestari, Armalya Pritazahra

**Affiliations:** 1 Department of Cardiology, Heart and Vascular Harapan Kita Hospital, Jakarta, Indonesia; 2 Department of Cardiology and Vascular Medicine, Faculty of Medicine, University of Indonesia, Jakarta, Indonesia; 3 Faculty of Medicine, University of Indonesia, Jakarta, Indonesia

## Abstract

Background: Drug-resistant tuberculosis remains a major public health challenge with substantial associated morbidity and mortality. Management requires complex, individualized treatment regimens. Emerging evidence suggests that contemporary antituberculosis agents for drug-resistant disease carry significant cardiotoxicity risk. This study aims to evaluate the cardiovascular toxicity of current therapeutic regimens in patients with multidrug-resistant, pre-extensively drug-resistant, and extensively drug-resistant tuberculosis.

Methods: This systematic review and meta-analysis was conducted following the PRISMA guidelines. A comprehensive literature search was performed across five databases (PubMed, Cochrane Library, Scopus, ProQuest, and Springer) through May 5, 2024. Methodological quality was assessed using NHLBU study quality assessment tools for controlled intervention studies. Statistical analyses were conducted using a random-effects model with the DerSimonian-Laird method, with results reported as pooled estimates with 95% confidence intervals.

Results: Eight studies comprising 9,506 patients with multidrug-resistant, pre-extensively drug-resistant, and extensively drug-resistant tuberculosis were included. Bedaquiline without delamanid was associated with the greatest QTc prolongation [pooled mean difference: 21.9 ms (95% CI [10.24–33.62])] and highest prevalence of clinically significant QTc prolongation [21.2% (95% CI [8.6–22.8]%)]. Treatment discontinuation rates were similar for bedaquiline monotherapy and bedaquiline-delamanid combination therapy [8% (95% CI [2–18]%) vs. 8% (95% CI [0–21]%)].

Conclusion: Drug resistant tuberculosis treatments were associated with cardiotoxicity concerns, evidenced by its QT prolongation, clinically significant arrhythmia, and treatment discontinuation.

## Background

Multi and extended drug-resistant (MDR and XDR) tuberculosis (TB) represent persistent global public health challenges with substantial associated morbidity and mortality. A systematic review by Salari et al. (2023) estimated pooled global prevalence rates of 11.6% for multidrug-resistant and 2.5% for extensively drug-resistant tuberculosis^1^. Treatment success rates for drug-resistant tuberculosis remain suboptimal, falling considerably short of the World Health Organization (WHO) target of 75%^2^. Management of drug-resistant tuberculosis necessitates complex, individualized therapeutic regimens. Current WHO consolidated guidelines recommend a 6-month regimen comprising bedaquiline, pretomanid, linezolid, and moxifloxacin (BPaLM) for multidrug-resistant and pre-extensively drug-resistant tuberculosis, while extensively drug-resistant tuberculosis requires an 18-month individualized regimen incorporating at least four effective agents^3,4^.

Bedaquiline, a novel diarylquinoline antimycobacterial agent, has demonstrated significant efficacy in the treatment of multidrug-resistant and extensively drug-resistant tuberculosis. Recent evidence indicates that bedaquiline is associated with shortened treatment duration and improved treatment success rates^5,6^.

Delamanid has been approved by some countries to use as part of longer individualized regimen for MDR TB^7^. However, a major concern with these treatment regimens is the numerous possible side effects, especially the troubling issue of cardiotoxicity which leads to treatment discontinuation.

Some studies have shown that the treatment of drug-resistant tuberculosis, including with bedaquiline, delamanid, and other cardiotoxic drugs, may cause QT prolongation^8^. QT prolongation heightens the risk of developing Torsades de Pointes, a potentially fatal arrhythmia^9,10^. Therefore, this study aims to evaluate the cardiotoxicity effect of recent treatment available in MDR, Pre-XDR, and XDR TB patients.

## Methods

This systematic review and meta-analysis was conducted according to Preferred Reporting Items for Systematic Reviews (PRISMA) guidelines.

### Search strategy and study selection

Two authors (KK and HLM) were equally assigned to perform literature search comprehensively through five databases (PubMed, Cochrane, Scopus, ProQuest, and Springer) up to May 5, 2024. The complete search strategy is provided in Supplement 1. Additionally, handsearching through article references of related studies was also performed to identify potential additional studies. After eliminating duplicate articles, all authors independently reviewed the titles and abstracts from the initial search. Any discrepancies were discussed through consensus. All study selection process were performed following this PICO framework: (1) Type of study: cohort study; (2) Population: extended and multi-drug-resistant tuberculosis drugs (XDR and MDR); (3) Intervention or exposure: Bedaquiline regimen; (4) Comparison: Any interventions; (5) Outcomes: QTcF interval, discontinuation of drugs due to prolong QT, and clinically significant arrhythmia. Studies were excluded under the following conditions: experimental (non-human) study, inaccessible full-text, conference paper, follow-up <6 months, and languages other than English or Indonesian.

### Data extraction and analysis

Two authors (KK and HLM) independently extracted data from included studies according to the Cochrane Handbook for Systematic Reviews of Interventions. The remaining authors checked the included studies. Any discrepancies were resolved by consensus. The following information was extracted from included studies: Study information (design, location, follow-up period); Participants baseline characteristics (number of subjects, gender, Tuberculosis status); Intervention information; Comparison (if any); and Outcomes (QTcF interval, rate of BDQ discontinuation due to prolong QT, and clinically significant arrhythmia). According to the International Council for Harmonisation (ICH) guideline, the definition of clinically significant QT prolongation is QTcF > 500 ms, or 60 ms increase from the baseline.

### Statistical analysis

Statistical analysis was performed using Review Manager 5.4 (Cochrane Collaboration, Oxford, UK) and R studio. For single arm dichotomous data, the proportional meta-analysis was performed. Meta-analysis of single means was used to calculate overall mean for single arm continuous data. Similarly, odds ratio and mean difference were used to calculate size effect for double arm data. All analysis was performed using the random-effects model and DerSimonian-Laird model, reported in 95% of confidence intervals (95%CI). Statistical heterogeneity was evaluated with I^2^ statistics. As outlined in the Cochrane Reviews Handbook, the I^2^ values were categorized as follows: 0–40% indicating no heterogeneity, 30–60% indicating moderate heterogeneity, 50–90% indicating substantial heterogeneity, and 75–100% indicating considerable heterogeneity. Publication bias was examined using a funnel plot.

### Quality assessment

Each study quality was assessed by two authors (KK and HLM) individually according to NHLBI study quality assessment tools for controlled intervention studies that consisted of 14 items. Any discrepancies were resolved by consensus or voting with the remaining authors. Judgements were ‘Good’, ‘Fair’, or ‘Poor’.

## Results

### Search results

**Figure 1.**
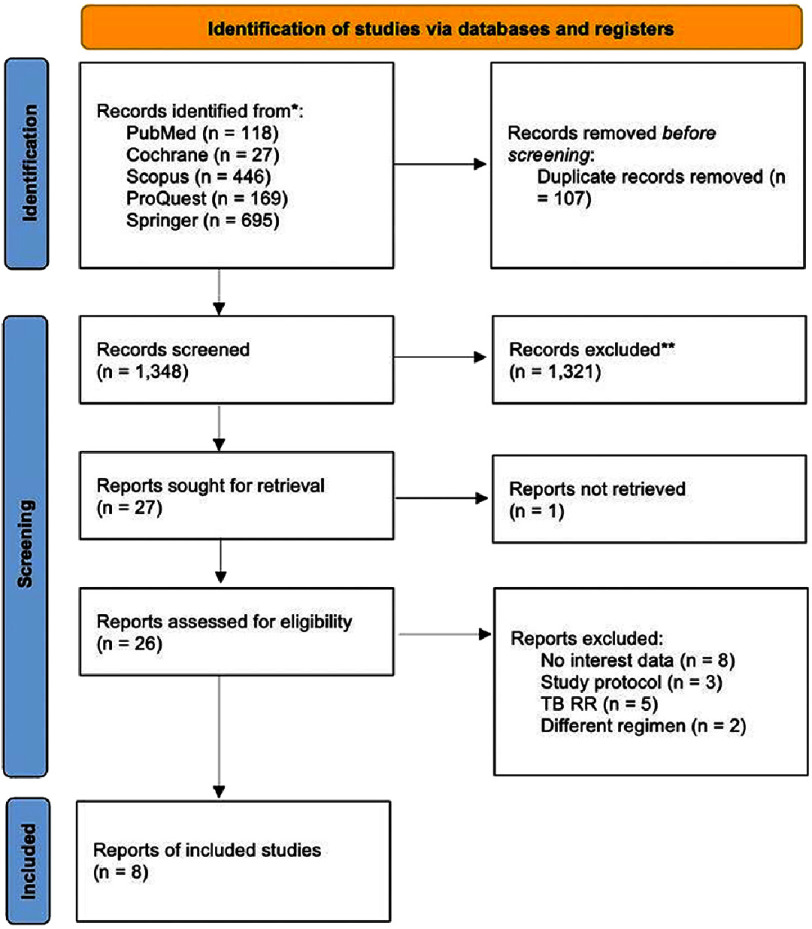
PRISMA flowchart.

A total of 1,455 articles were retrieved during the initial search. After deduplicating, title and abstract screening were performed in 1,348 articles. The authors further excluded 1,321 due to irrelevant articles. One more article was also excluded due to being unable to retrieve the article. Simultaneously, full-text screening was performed through the remaining 26 articles for eligibility. Ultimately, 8 studies fulfilled our inclusion criteria and were included in the analysis. The PRISMA flowchart was depicted in Figure 1.

### Included studies

A total of 8 studies were eligible for this systematic review and meta-analysis [Ferlazzo et al. (2018), Sarin et al. (2019), Gao et al. (2021), Darmayani et al. (2022), Pai et al. (2022), Kim et al. (2023), Li et al. (2023), and Jin et al. (2023)]. Five out of eight studies were conducted in Asia, two in Africa, and one in Europe. Table 1 shows the baseline characteristics of the included studies.

**Table 1:** Baseline characteristics of included studies.

No	Author (Year)	Study design	Location	Follow-up period	Study population		Intervention	Comparison
					Subjects (M/F)	Category of DR-TB (N/%)		
1.	Ferlazzo et al. (2018)^11^	Retrospective cohort	South Africa	6 months	28 (17/11)	MDR-TB (2/7.1%) Pre-XDR-TB FQ (10/36%) Pre-XDR-TB injectable (2/7%) XDR-TB (14/50%) HIV Co-infection (11/28%)	Bdq+Dlm + Lzd (23/82%) + Cfz (19/68%) + Mfx (6/21%) + Carbapenem (15/54%)	N/A
2.	Sarin et al. (2019)^12^	Retrospective cohort	India	6 months	53 (24/29)	MDR-TB (35/67%) Pre-XDR-TB (1/2%) XDR-TB (17/32%) HIV Co-infection (0/0%)	– Bdq + Dlm + Imp (21/40%) – Bdq + Dlm + Imp + Mfx (42/79%) – Bdq + Dlm + Mfx (31/58%) – Bdq + Dlm + Imp + Mfx + Lzd + Cfz (22/42%)	N/A
3.	Gao et al. (2021)^13^	Prospective cohort	China	6 months	1162 (813/349)	MDR-TB (382/32.9%) Pre-XDR-TB (492/42.3%) XDR-TB (288/24.8%) HIV Co-infection (NR)	Bdq + at least 4 drugs without Dlm (1162) Mfx, Lfx, Lzd, Cfz, Am, Cm, Pto, Cs, Z, E, PAS, Hh, Mpm. and Amx-CLv Cfz (694/59.7%) Lfx (157/13.5%) Gatifloxacin (1/0.1%)	N/A
4.	Darmayani et al. (2022)^14^	Retrospective cohort	Indonesia	6 months	105 (61/44)	MDR (79/75.2%) Pre-XDR (18/17.1%) XDR (8/7.6%) HIV Co-infection (0/0%)	- BDQ only (2) - Two drugs: Lfx/BDQ (20); Mfx/BDQ (3); BDQ/Cfz (14) - Three drugs: Lfx/BDQ/Cfz (51); Mfx/BDQ/Cfz (14) - Four drugs: Lfx/Bdq/Cfz/Dlm (1)	N/A
5.	Pai et al. (2022)^15^	Retrospective cohort	South Africa	30 months	5981 (3358/2623)	MDR-TB (3327/55.6%) Pre-XDR-TB (1251/20.9%) XDR-TB (1403/23.5%) HIV Co-infection (4271/71.4%)	Bdq-containing regimen without Dlm (3747) According to 2011 and 2017 South Africa Guideline Cfz (3016/50.4%) Mfx (3193/65.4%)	Non-Bdq-containing regimen (2234)
6.	Kim et al. (2023)^8^	Retrospective cohort	South Korea	36 months	1998 (1354/644)	MDR-TB HIV Co-Infction (NR)	Bdq + at least 3 second-line anti-TB drugs (315) Fluoroquinolones (1813/0,91) Clofazimine (0/0%)	– Dlm-containing regimen (292) – Conventional regimen (1391)
7.	Li et al. (2023)^16^	Retrospective cohort	China	6 months	85 (54/31)	MDR-TB	Group A: Bdq containing regimen without other qt prolong drug (33)	Group B: Bdq + FQ and/or Cfz (52) Cfz (20) Cfz + FQ (12) FQ (20)
8.	Jin et al. (2023)^17^	Prospective cohort	Georgia	12 months	94 (77/17)	MDR or XDR-TB	Bdq with Cfz/Lfx/Mfx	Dlm with Cfz/Lfx/Mfx

### Participants

A total of 9,506 extended, pre-extended, and multi-drug resistant Tuberculosis patients were included in this study. Two studies [Kim et al. (2023) and Li et al. (2023)] only included MDR-TB patients. However, XDR-TB patients were included in other studies. 

### Intervention

All participants received individualized anti-tuberculosis treatment regimens according to the WHO and national guideline, as well as their antibiotic resistance profiles. Ferlazzo et al. (2018) and Sarin et al. (2019) used regimens consisting of a combination of bedaquiline and delamanid, in addition to other drugs. The bedaquiline regimen without delamanid was used in other remaining studies. Three studies [Pai et al. (2022), Kim et al. (2023), and Jin et al. (2023)] compared bedaquiline to non-bedaquiline regimen (including delamanid).

### Outcomes

The detailed outcomes are listed in Table 2.

**Table 2:** Outcomes of included studies.

**No**	**Author (Year)**	**Mean QTcF different (SD)**	**Discontinuation of BDQ**	**Clinically Significant QT Prolongation**
1.	Ferlazzo et al. (2018)^11^	– Baseline QTcF: 401 (381–432) 6m QTcF: 434 (408–446) – Median change: 16 (−13–31) – Estimated mean: 11.0 ± 34.37	N/A	Definition: QTcF > 500 ms or increase > 60 ms from baseline. 1/28 (0.04%)
2.	Sarin et al. (2019)^12^	N/A	4/53 (7.5%)	Definition: QTcF > 500 ms or increase > 60 ms from baseline. 11/53 (20%)
3.	Gao et al. (2021)^13^	– Baseline QTcF: 413 (398–429) – 6M median change: 16 (−3–35) – Estimated mean: 16 ± 28.2	49/1162 (4.2%)	Definition: QTcF > 500 ms 287/1162 (24.7%)
4.	Darmayani et al. (2022)^14^	Baseline QTc: 414.52 ± 33.74 Mean QTcF different: 23.97 (52.82) *p* < 0.011 (5.83-42.11)	16/52 (15.2%)	Definition: QTcF > 500 ms or increase > 60 ms from baseline. 39/105 (37.1%)
5.	Pai et al. (2022)^15^	N/A	BDQ: 45/3747 (1.2%)	Definition: QTcF > 500 ms BDQ vs non-BDQ: 179/3747 (4.8%) vs. 5/2234 (0.2)
6.	Kim et al. (2023)^8^	N/A	N/A	Definition: QTcF > 500 ms BDQ vs Dlm vs Conventional: 5/313 (1.6%) vs 8/286 (2.8%) vs 23/1372 (1.7%)
7.	Li et al. (2023)^16^	**Group A** – Baseline: 401.06 (17.96) – 6m: 418.56 (19.49) – Mean difference: 17.5 ± 2.875 ** Group B** – Baseline: 402.42 (19.72) – 6m: 433.11 (30.81) – Mean difference: 30.69 ± 5.07	0	Definition: QTcF > 500 ms Group A vs. B: 3/33 (9.1%) vs. 18/52 (34.6%) OR A vs B: 0.26 (0.07-0.96), *p* = 0.04
8.	Jin et al. (2023)^17^	Mean QTcF different 6m Bdq vs Dlm: 16.33 ± 22.91 vs 19.75 ± 50.53	N/A	Definition: QTcF > 500 ms Bdq vs Dlm: 9/30 vs 14/64

**Figure 2.**
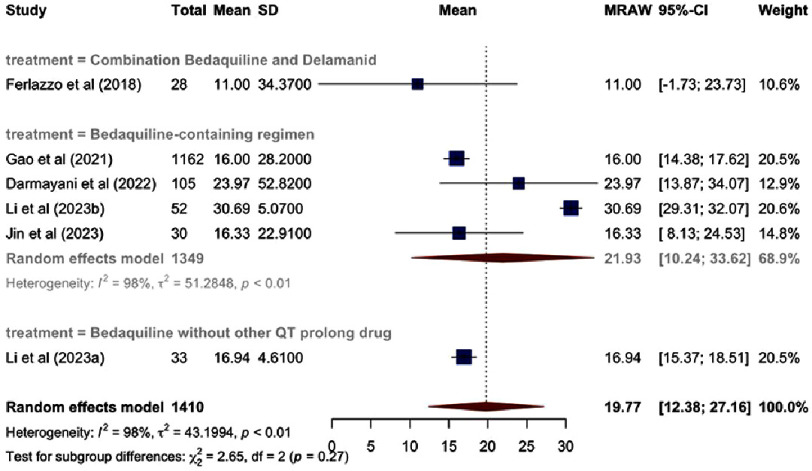
Mean QT interval changes during bedaquiline treatment.

**Figure 3.**
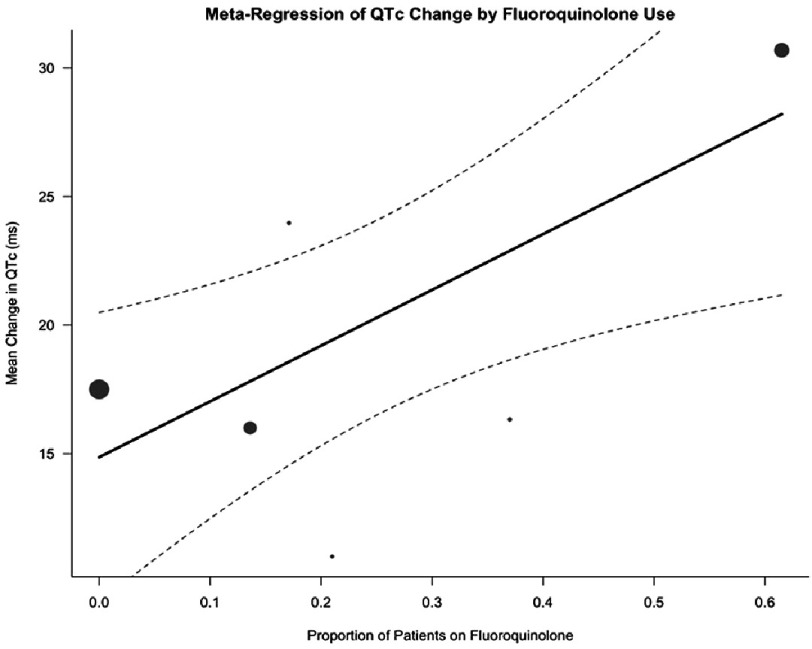
Meta-regression bubble plot of QTc change by fluoroquinolone use.

### Patient QT interval profiles

Five studies reported mean QTc interval changes during bedaquiline therapy. Figure 2 presents the pooled mean QTc change stratified by treatment regimen. Although the pooled mean QTc prolongation in the bedaquiline-containing regimen subgroup (mean difference: 21.93 ms, 95% CI [10.24–33.62]) was numerically greater than in the bedaquiline-delamanid combination subgroup (mean difference: 11.00 ms, 95% CI [−1.73–23.73]), this difference did not achieve statistical significance (test for subgroup differences, *P* = 0.27). Substantial heterogeneity was observed across studies (I^2^ = 98%), as illustrated in Figure 2.

To identify sources of heterogeneity, multivariable meta-regression was performed incorporating concomitant clofazimine and fluoroquinolone use as moderator variables. This model accounted for 79.4% of the observed heterogeneity (*R*^2^ = 79.4%), with residual heterogeneity no longer statistically significant (*I*^2^ = 41.2%, *P* = 0.13). Higher proportions of patients receiving fluoroquinolones were significantly associated with greater QTc prolongation (*β* = 28.70, *P* = 0.0006), as depicted in Figure 3, whereas clofazimine use was not a significant predictor (*P* = 0.21).

**Figure 4.**
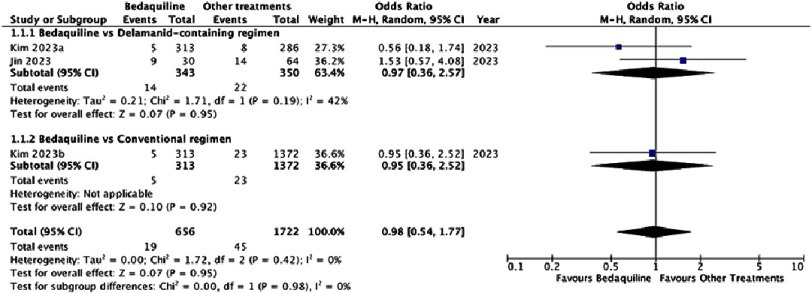
Pooled odds ratio of clinically significant QT prolongation.

**Figure 5.**
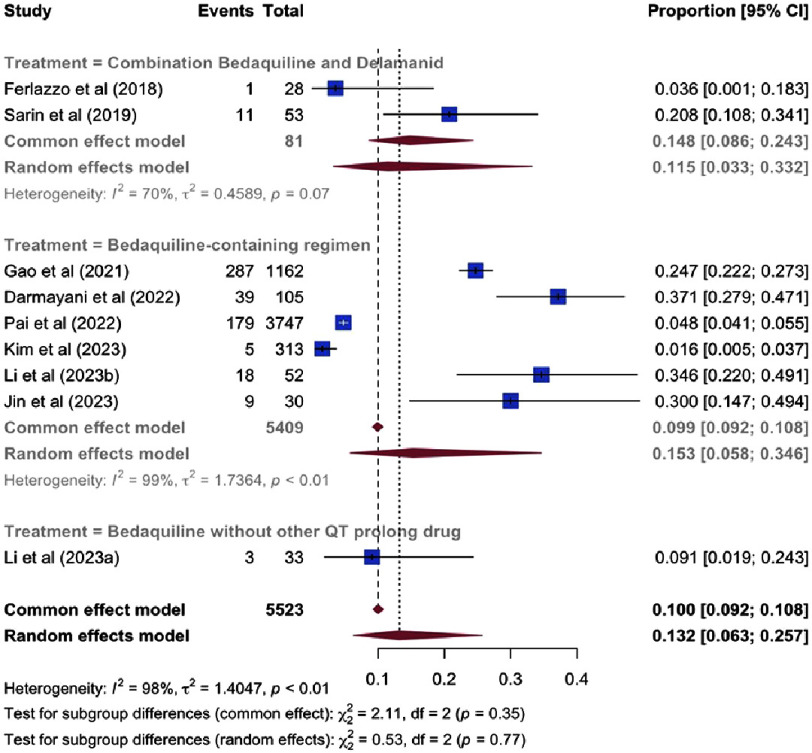
Pooled prevalence of clinically significant QT prolongation (using Logit transformation).

**Figure 6.**
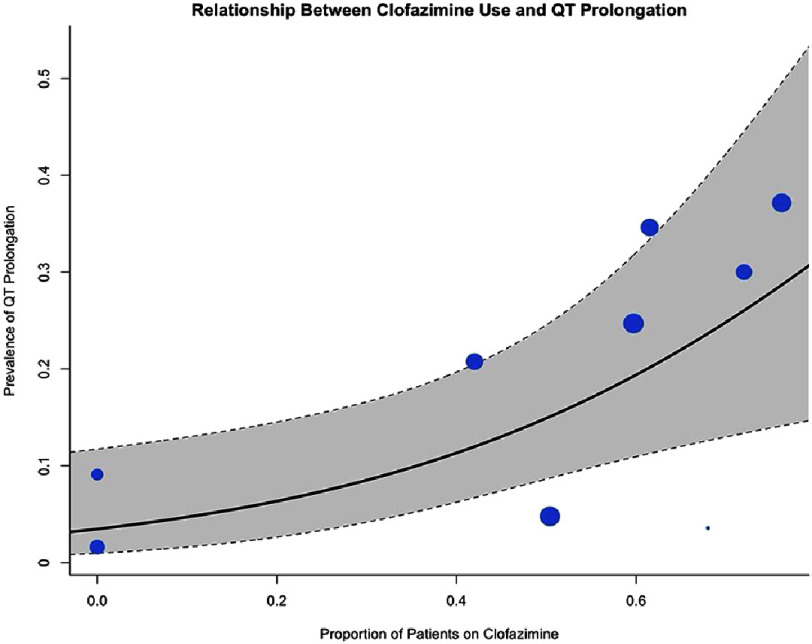
Meta-regression bubble plot of clinically significant QT prolongation by clofazimine use.

**Figure 7.**
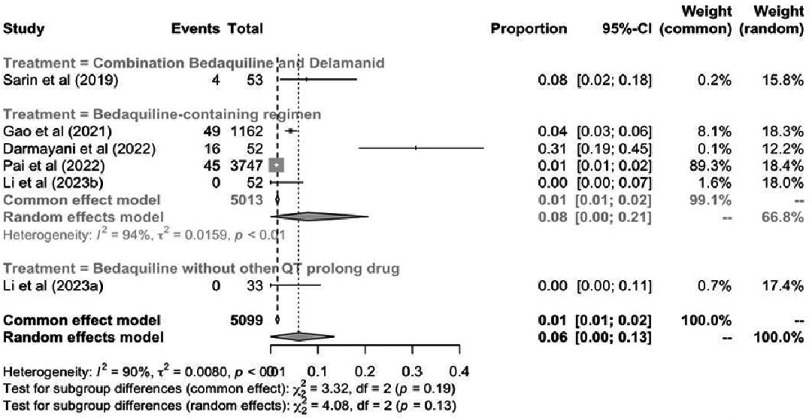
Pooled prevalence of treatment discontinuation due to cardiotoxicity.

### Clinically significant QT prolongation

The risk of clinically significant QTc prolongation in patients receiving bedaquiline-containing regimens was comparable to that observed with delamanid-containing regimens [odds ratio (OR): 0.97, 95% CI [0.36–2.57], *P* = 0.95] and conventional regimens [OR: 0.95, 95% CI [0.36–2.52], *P* = 0.92], as illustrated in Figure 4. The pooled prevalence of clinically significant QTc prolongation among patients receiving bedaquiline was 13.2% (95% CI [6.3–25.8]%, I^2^ = 98%), as shown in Figure 5.

Given the substantial heterogeneity, multivariable meta-regression was performed to identify potential sources of between-study variance. The analysis, incorporating 9 studies, demonstrated overall model significance (*Q* = 7.35, *P* = 0.025) and accounted for approximately 49.4% of the observed heterogeneity (*R*^2^ = 49.4%). Higher proportions of patients receiving concomitant clofazimine emerged as a significant predictor of increased QTc prolongation prevalence (*β* = 2.86, *P* = 0.025), as depicted in Figure 6. Conversely, the proportion of patients receiving fluoroquinolones was not a statistically significant moderator after adjustment for clofazimine use (*P* = 0.42).

### Treatment discontinuation due to QT prolong

Figure 7 demonstrates that the prevalence of treatment discontinuation was comparable between patients receiving bedaquiline without delamanid and those receiving bedaquiline-delamanid combination therapy [8% (95% CI [2–18]%) vs. 8% (95% CI [0–21]%), respectively]. Notably, Li et al. (2023) reported no treatment discontinuation in patients receiving bedaquiline without other QTc-prolonging agents.

### Publication bias

The authors were unable to examine publication bias due to lack of studies minimum (10 studies) for meta-analysis.

### Risk of bias

The quality assessment of included studies are depicted in Supplementary 2. The authors used NHLBI study quality assessment tools for intervention studies. Five out of eight studies were rated as good quality, the three remaining were fair. Most studies did not report sample size justification. Besides, three rated fair studies, which were Darmayani et al. (2023), Sarin et al. (2019), and Ferlazzo et al. (2018), did not measure or adjust for potential cofounding variables.

## Discussion

### QT prolongation in patients with tuberculosis

The QT interval represents the duration of ventricular depolarization and repolarization. Drug-induced QT prolongation, particularly associated with bedaquiline, delamanid, clofazimine, and fluoroquinolones, is predominantly mediated through inhibition of the rapidly activating delayed rectifier potassium current (I_Kr_), which is critical for cardiac repolarization. This current is conducted through potassium channels encoded by the human ether-à-go-go-related gene (*hERG*)^11–13^.

### Summary of findings

To our knowledge, this represents the first meta-analysis specifically evaluating cardiovascular toxicity associated with antituberculosis treatment regimens in patients with multidrug-resistant, pre-extensively drug-resistant, and extensively drug-resistant tuberculosis. Our findings demonstrate that QTc interval prolongation occurred across all treatment groups, regardless of regimen composition.

Patients receiving bedaquiline without delamanid demonstrated the greatest QTc interval prolongation compared to alternative regimens [pooled mean difference: 21.9 ms (95% CI [10.24–33.62])]. The substantial heterogeneity observed was largely attributable to concomitant use of other QTc-prolonging agents, particularly fluoroquinolones and clofazimine, which varied across included studies. Fluoroquinolones, widely employed as second-line antituberculosis agents, are well-established causes of QTc prolongation^14,15^. Similarly, clofazimine, extensively utilized in drug-resistant tuberculosis regimens, has been demonstrated to prolong the QTc interval, particularly when combined with other proarrhythmic agents. Abdel wahab et al. (2021) reported a dose-dependent relationship between clofazimine and QTc prolongation prevalence^16^. The greatest mean QTc change was observed in the study by Li et al (2023), which incorporated three QTc-prolonging agents—bedaquiline, fluoroquinolone, and clofazimine—yielding a mean difference of 30.69 ms (95% CI [29.31–32.07]).

Two studies, Kim et al. (2023) and Jin et al. (2023), directly compared bedaquiline-containing regimens to alternative treatments^8,17^. Our analysis revealed no statistically significant difference in clinically significant QTc prolongation between bedaquiline-containing regimens and either delamanid-containing regimens [OR: 0.97 (95% CI [0.36–2.57])] or conventional regimens [OR: 0.95 (95% CI [0.36–2.52])]. However, single-arm studies demonstrated higher prevalence rates of clinically significant QTc prolongation with bedaquiline-containing regimens. Notably, Li et al. (2023) reported significantly lower incidence of clinically significant arrhythmia in patients receiving bedaquiline without other QTc-prolonging agents compared to those receiving combination therapy with bedaquiline, fluoroquinolones, and clofazimine [OR: 0.26 (95% CI [0.07–0.96])]^18^.

These findings are consistent with a systematic review by Tong et al 2023 evaluating bedaquiline safety in general tuberculosis populations, which demonstrated increased cardiotoxicity incidence with bedaquiline compared to non-bedaquiline regimens [relative risk: 4.54 (95% CI [1.74–11.87])]. However, the composition of non-bedaquiline comparator regimens was not specified in that analysis^19^.

Regarding treatment discontinuation attributable to cardiotoxicity, bedaquiline-containing regimens and bedaquiline-delamanid combination therapy demonstrated comparable discontinuation rates of 8%. Notably, Li et al. (2023) reported no treatment discontinuation in patients receiving bedaquiline without concomitant QTc-prolonging agents^18^. Similarly, Olaru et al. (2017) documented no bedaquiline-related treatment discontinuation due to adverse events^19^. A systematic review by Pontali et al. (2017) reported bedaquiline discontinuation in 3.5% of patients due to adverse events; however, the specific proportion attributable to QTc prolongation was not delineated^12^.

Our findings underscore the heterogeneity of cardiotoxicity risk associated with antituberculosis treatment regimens. Numerous patient-specific risk factors and clinical conditions are recognized contributors to QTc prolongation, including advanced age, female sex, hepatic or renal dysfunction, electrolyte disturbances, and inherited channelopathies such as congenital long QT syndrome. However, most included studies implemented rigorous exclusion criteria to mitigate these confounding variables and isolate drug-specific effects on QTc interval^17,18,20^.

### Clinical implications

Our findings demonstrate that bedaquiline, delamanid, and other QTc-prolonging antituberculosis agents are associated with QTc interval changes in patients with multidrug-resistant, pre-extensively drug-resistant, and extensively drug-resistant tuberculosis. Notably, bedaquiline monotherapy demonstrated a non-significant safety advantage compared to delamanid-containing or conventional regimens. However, bedaquiline regimens incorporating additional QTc-prolonging agents were associated with higher prevalence of clinically significant QTc prolongation and treatment discontinuation. Meta-regression analysis identified concomitant clofazimine and fluoroquinolone use as significant determinants of this heterogeneity. These findings suggest that while bedaquiline-based regimens represent a cornerstone of contemporary drug-resistant tuberculosis therapy, the concurrent administration of other QTc-prolonging agents—particularly clofazimine and fluoroquinolones—substantially amplifies the risk of clinically significant cardiac QT prolongation.

These findings may inform clinical guideline development and assist clinicians in optimizing treatment regimens for patients with drug-resistant tuberculosis. Although subgroup analysis revealed no statistically significant differences in pooled outcomes between major regimen categories, meta-regression analysis strongly indicates that heightened vigilance is warranted when multiple QTc-prolonging agents are administered concomitantly. We recommend implementation of baseline and serial electrocardiographic monitoring to ensure patient safety and facilitate treatment adherence throughout the therapeutic course^13,20,21^.

### Study limitations

Several limitations warrant acknowledgment. First, individual studies enrolled relatively small sample sizes, and treatment regimens varied considerably across studies, contributing to heterogeneity. Second, insufficient granular data precluded comprehensive analysis of the specific contributions of individual QTc-prolonging agents within bedaquiline-containing regimens. Finally, the absence of randomized controlled trials among included studies represents a deviation from the gold standard for intervention research, potentially introducing selection bias and limiting causal inference.

### Recommendations

Further studies with a large number of subjects and similar treatment using randomized controlled trials are warranted to prove the cardiotoxicity effect of drug-resistant TB treatment regimen.

## Conclusion

Treatment regimens for drug-resistant tuberculosis are associated with significant cardiotoxicity concerns, manifesting as QTc interval prolongation, clinically significant arrhythmias, and treatment discontinuation.

## Conflicts of interest

The authors have declared that there are no conflicts of interest.

## Author contributions

**LDL** proposed study concept and design.

**KK and HFL** performed data acquisition, analysis and interpretation of data, and drafting the manuscript.

All authors (**LDL, KK, HFL, and AP**) have performed critical review of the manuscript.
